# The application of selected radionuclides for monitoring of the D–D reactions produced by dense plasma-focus device

**DOI:** 10.1007/s10967-014-3131-0

**Published:** 2014-04-17

**Authors:** S. Jednorog, A. Szydlowski, B. Bienkowska, R. Prokopowicz

**Affiliations:** 1 Institute of Plasma Physics and Laser Microfusion EUROATOM Association, Hery Street 23, 01-497 Warsaw, Poland; 2 National Center for Nuclear Reseach, Andrzeja Soltana Street 7, 05-400 Otwock, Swierk, Poland

**Keywords:** Dense plasma-focus, Neutron activation diagnostics, Silver activation counter, Fusion neutrons yttrium monitor, Beryllium neutron activation counter, Indium neutron distribution monitor

## Abstract

The dense plasma focus (DPF) device—DPF-1000U which is operated at the Institute of Plasma Physics and Laser Microfusion is the largest that type plasma experiment in the world. The plasma that is formed in large plasma experiments is characterized by vast numbers of parameters. All of them need to be monitored. A neutron activation method occupies a high position among others plasma diagnostic methods. The above method is off-line, remote, and an integrated one. The plasma which has enough temperature to bring about nuclear fusion reactions is always a strong source of neutrons that leave the reactions area and take along energy and important information on plasma parameters and properties as well. Silver as activated material is used as an effective way of neutrons measurement, especially when they are emitted in the form of short pulses like as it happens from the plasma produced in Dense Plasma-Focus devices. Other elements such as beryllium and yttrium are newly introduced and currently tested at the Institute of Plasma Physics and Laser Microfusion to use them in suitable activation neutron detectors. Some specially designed massive indium samples have been recently adopted for angular neutrons distribution measurements (vertical and horizontal) and have been used in the recent plasma experiment conducted on the DPF-1000U device. This choice was substantiated by relatively long half-lives of the neutron induced isotopes and the threshold character of the ^115^In(n,n′)^115m^In nuclear reaction.

## Introduction

The nuclear synthesis of light element is considered to be the promising source of energy in the near future. The process of the controlled nuclear fusion is very complex and needs to monitor a lot of parameters that are crucial for plasma operations. During large plasma experiments huge fluxes of neutrons are produced and then leave the plasma. These neutrons carry away the released reaction energy as well as important information on plasma parameters, and especially on fusion reaction reagent characteristics. The dense plasma-focus (DPF) device named DPF-1000U is operated at the Institute of Plasma Physics and Laser Microfusion (IPPLM). It generates a dense and high-temperature plasma which is a powerful source of neutrons. These neutrons are monitored and diagnosed with the use of different techniques. Recently a special stress has been laid in our institute on the development and upgrading of the activation method. Radionuclides that are generated during interactions of neutrons with target materials are the important source of information about the plasma generated in DPF-1000U device as well as neutrons accompanying this phenomenon. The isotopes of silver, yttrium, indium and beryllium are considered as important tool for plasma monitoring.

## Plasma formatting by means of DPF-1000U device

The DPF-1000U is at the moment the world’s largest Plasma-Focus machine operating with deuterium as a working gas. It produces pulsed, short-lived (~100–200 ns) plasma pinches with plasma temperature approaching 1 keV and density of the order of 10^19^ cm^−3^. The development of MHD instabilities of the pinch column as well as various types of micro-instabilities leads to generation of intense streams of relativistic electrons and fast ions. Plasma-Focus machines are known as the very intense, pulsed sources of fast neutrons (~2.45 MeV) originated from D–D fusion reactions. Neutrons of the beam-target nature (accelerated deuterons – hot deuteron plasma) are emitted simultaneously with neutrons of the thermonuclear origin. It is estimated that beam-target neutrons represent the majority of all generated neutrons, even up to 90–95 % of all of them.

The DPF-1000U machine, operating at the IPPLM is capable of emitting mainly 5·10^11^ neutrons/pulse [1]. Because of that the DPF-1000U has been intended for basic studies of hot plasma phenomena. This device is also very useful for testing and calibrating different diagnostic equipment. This is mainly due to the intensive neutron and X-ray generation from DPF discharges. The diagnostic tools which have been calibrated in this way are afterward installed on other larger plasma facilities, such as tokamaks, laser systems etc.

**Fig. 1:**
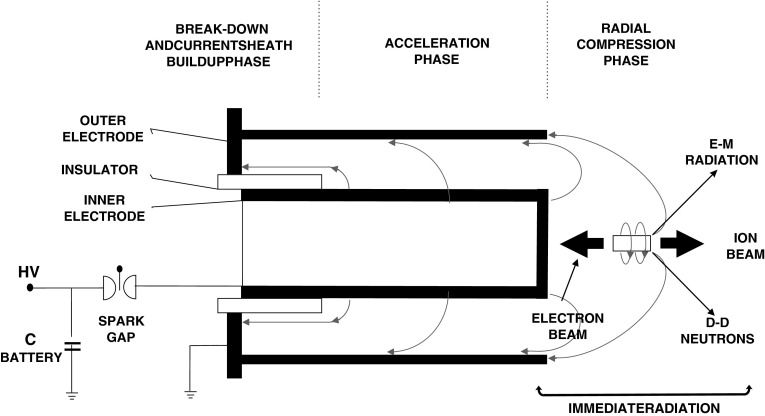
The schematic view of plasma production at DPF-1000U device; where: *V*_z_,*V*_r_ axial and radial velocity of current sheath respectively, *J* electric current flowing through the plasma focus, *B*_Φ_ azimuthal magnetic field

The vacuum chamber of DPF-1000U device has the shape of a large cylinder with walls made of stainless steel. The DPF-1000U device is equipped with a large condenser battery with the capacity of 1,332 μF. The battery can be charged up to 40 kV that results in 1 MJ of energy stored in the battery. However the discharges are usually performed with the voltage of 24 kV, which is equivalent to 400 kJ. The discharge circuit consists of a set of cables and spark-gaps which connect the capacitor battery with cylindrical concentric electrodes playing the role of a plasma accelerator, which is sometimes called a plasma gun. Figure 1 presents a schematic view of the DPF-1000U device and phases of plasma formation at the above-mentioned device.

The electrode assembly is located inside the vacuum chamber and is composed of three units; (1) a tubular anode (inner electrode), (2) a ceramic insulator embracing the anode at one end, and (3) a cathode composed as the outer electrode of the gun. The anode is made of a copper tube and has a diameter of 23 cm and a length of 48 cm. The cathode is composed of 12 stainless steal rods with a diameter of 5 cm each, which are symmetrically distributed on a cylinder surface with a diameter equal to 40 cm.

When the vacuum vessel is filled with deuterium (D_2_), the discharge current that flows through the electrodes reaches an intensity of 2 MA and the so-called current sheath is formed. It is formed along the insulator surface (break-down and current sheath build up phase: see Fig. 1). Then the Lorentz’s force tears away the sheath and accelerates it along the anode axis (Acceleration phase: see Fig. 1). During this discharge phase the gaseous deuterium gathered in the space between the electrodes is pushed towards the open electrode end. In the last phase of the discharge the current sheath collapses and is accelerated radially inwards (radial compression phase: see Fig. 1), and so-called dense plasma focus (pinch) is created. The density and temperature of the plasma in the focus phase are so high that they are favourable for the nuclear fusion. Then, the induced plasma emits a large pulse of neutrons up to 10^12^ neutrons/pulse (mainly 5·10^11^ neutrons/pulse). These neutrons have energy of around 2.5 MeV and escape from the plasma focus in all directions.

## Nuclear reaction occurring in plasmas generation on DPF-1000U

The dense and hot deuterium plasma which is created in the last phase of the DPF discharge is a source of the following nuclear fusion reactions:

1 $$ \begin{aligned} & {\text{D }} + {\text{ D}} \to\,^{3}\!{\text{He}}\left( {0. 8 2 {\text{ MeV}}} \right) \, + {\text{ n }}\left( { 2. 4 5 {\text{ MeV}}} \right);     \quad R_{a} \approx { 5}0\;\% , \\ & {\text{D }} + {\text{ D}} \to {\text{T }}\left( { 1.0 1 {\text{ MeV}}} \right) \, + {\text{ p }}\left( { 3.0 2 {\text{ MeV}}} \right);\quad         R_{a} \approx { 5}0 \, \% , \\ & {\text{D }} + {\text{ D}} \to\,^{ 4}{\text{He }} + \, \gamma \, \left( { 2 3. 8 {\text{ MeV}}} \right);\quad                           R_{a} \approx { 1}0^{ - 5}\, \% , \\ \end{aligned} $$

where *R*_a_ is the particular nuclear reaction abundance.

The kinetic energies of thermonuclear reaction products are mentioned in the brackets.

The total neutron yield (*Y*_n_) depends on various parameters of the discharge in the DPF-1000U device. Among them, the most important are: the deuterium gas pressure, the energy stored in the battery, the battery voltage, the geometry of the discharge and so on. These parameters and conditions could vary from discharge to discharge that results in fluctuations of neutron streams emitted in a particular discharge called pulses and sometimes shots due to the plasma acceleration phenomenon.

## Plasmas diagnostics based upon chosen nuclear reaction

Neutrons as a product of the nuclear fusion reaction easily escape from hot plasmas and they carry the important information on plasma parameters and fusion reaction mechanisms. Therefore, the neutron diagnostics are essential in estimating these parameters as well as released fusion power. Activation techniques are especially advantageous for the estimation of some characteristics of fast neutrons emitted from hot plasma discharges and have been used in many tokamak experiments [2]. These techniques were used to measure e.g. neutron fluence at chosen locations around the machine, even inside the vacuum vessel close to the vessel walls  [3]. Supported by neutron transport calculations the activation technique can provide information on the *Y*_n_ and released fusion power. The activation technique is the off-line, remote, and time integrated method. It does not interfere and interrupt plasma parameters like temperature, density and purity either.

### Activation technique, MCNP calculation and calibration procedures

The neutron activation technique is based on measurements of the activity induced by neutrons in particular sample and after that the determination of *Y*_n_. Because the emission of the neutrons from DPF-1000U discharge is short comparing to other processes, the initial activity induced in a sample material is expressed by the following formula:

2 $$ \begin{aligned} A_{0} &= \lambda \,N_{T} \int\limits_{0}^{E} {\sigma (E_{n} )\, \cdot }\, \varphi (E_{n} ) \cdot dE_{n} = \lambda \,N_{T} \left\langle {\sigma \cdot \varphi } \right\rangle , \hfill \\ N_{T} &= \frac{{m \cdot N_{A} }}{A}, \hfill \\ \end{aligned} $$

where *λ* is the decay constant, *N*_*T*_ number of nuclei in the sample, *σ*(*E*_n_) nuclear reaction cross-section, *φ*(*E*_n_) neutron flux density in the sample, *E*_n_ neutrons energy, E neutrons energy range, m sample mass, N_A_ Avogadro number, A sample atomic number, <σ·φ> nuclear reaction rate. The above mentioned integral named as the nuclear reaction rate can be therefore obtained from measured activity.

All experimental conditions like geometry of the device, geometry of the detector and plasma pinch as the source of neutrons are implemented in the Monte Carlo transport code. As a result, nuclear reaction rate <σ·φ>_N_ normalized per one neutron from the source is obtained. The ratio of the nuclear reaction rate obtained from the measured activity of the sample to the nuclear reaction rate calculated by Monte Carlo transport code gives the *Y*_n_ for DPF 1000U device:

3 $$ \frac{{\left\langle {\sigma \cdot \varphi } \right\rangle }}{{\left\langle {\sigma \cdot \varphi } \right\rangle_{N} }} = Y_{n}. $$

Currently applied calibration procedures are different than that carried many years ago. Numerical simulation plays the main role. Its result in comparison with experimental calibration data confirms the proper construction of the calculation input and an assumption used for neutron simulation.

Activation technique based on different radio-nuclides and used as the neutron yield monitor needs different calibration procedure. It dependents on the role which particular monitor will play in the device steering, controlling, monitoring etc.

Some of methods listed below need to be calibrated both by neutron source and particle simulation code. The silver activation counter (SAC) that has been used since many years as the *Y*_n_ monitor for the DPF-1000U device was calibrated by neutron source many times. Since the calibration procedure is costly and needs special preparation and precaution it is possible to complete this calibration no more frequently than once every dozen years, nevertheless those needs are different. The next neutron source calibration (NSC) of SAC will be combined with MCNP calculation. The SAC method has many limitations and because of that it will be operated only until the full implementation of other methods of *Y*_n_ monitoring.

The beryllium neutron activation counter (BNAC) is a good example of contemporary *Y*_n_ monitoring method. It will be in use simultaneously with SAC for a short period of time, probably a few years after next NSC of the DPF-1000U device. After the withdrawal of SAC it will be one of the main methods for *Y*_n_ monitoring.

The fusion neutron yttrium monitor (FNYM) is on the early phase of its implementation as the *Y*_n_ monitor. As the preliminary experiment shows, the linear dependence of yttrium radioactivity in the dependence of *Y*_n_ it is promising to use yttrium radionuclide as the neutron monitoring. In this phase the radioactivity determination needs careful efficiency calibration of the gamma spectrometers that was used for radioactivity measurement. It was ensured by both the numerical characteristic of the detector and its mathematical efficiency calibration. The next generation of the FNYM will need for its full implementation NSC, MCNP calculation and mathematical efficiency calibration of the gamma spectrometry system used for its radioactivity determination.

Indium radio-nuclides play an important role in large plasma experiment. In DPF-1000U device radio-indium was dedicated for the determination of the horizontal and vertical distribution of neutrons only. Because only qualitative information regarding neutron angular distribution was sought the monitor was not calibrated in any special way. Only careful measurement of ^115m^In activity was performed and special massive indium sample set was used as the threshold neutron monitor. That allowed the measurement of neutron anisotropy.

### Silver activation monitor

Naturally occurring silver is composed of two stable isotopes, ^107^Ag and ^109^Ag, with ^107^Ag being slightly more abundant (51.839 % natural abundance).

**Fig. 2:**
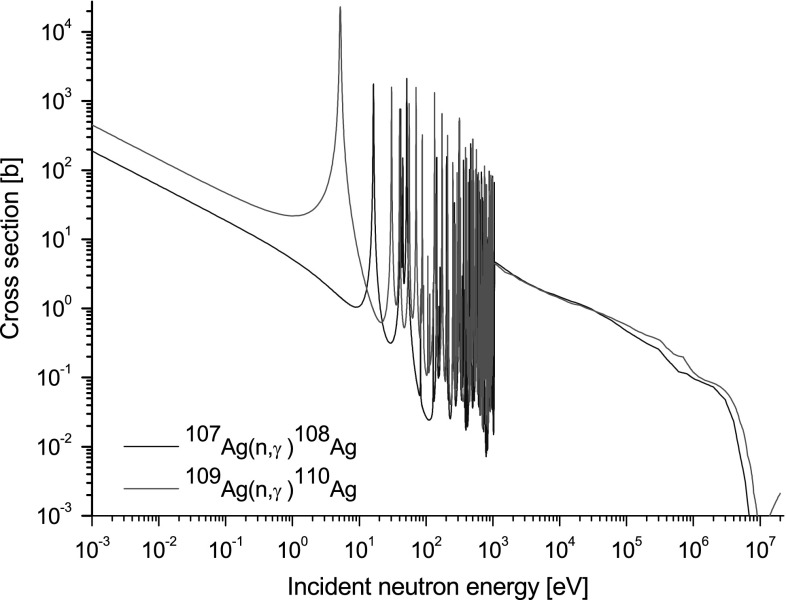
Cross-sections for radiative captures of neutrons by ^107^Ag (*blue line*) and ^109^Ag (*red line*) from JEFF 3.1.2. (Color figure online)

A SAC used to monitor short neutron pulses from a plasma discharge has several principled virtues: (1) it preserves fluence information after the pulse, (2) it has relatively high sensitivity mainly due to the high nuclear reaction cross section values (see Fig. 2), especially in the low neutron energy range, (3) its dynamic range can span a few order of magnitude of neutron intensity depending mainly on its dimensions. Therefore, SAC has been used for many years in different high-temperature plasma experiments and especially in DPF facilities for measuring neutron yield form pulsed sources [4].

This detector is composed of a Geiger–Muller (G-M) counter wrapped with a silver foil and placed within a hydrogen-reach moderator. Neutrons emitted from a pulsed source are slowed down in the moderator and induce two radiative capture nuclear reactions (see Eq. 4) in the foil made of natural silver:

4 $$ \begin{aligned} & {\text{n }} +\,^{ 10 7}\!{\text{Ag}} \to\,^{ 10 8}\!{\text{Ag }}\left( {T_{\raise.5ex\hbox{$\scriptstyle 1$}\kern-.1em/ \kern-.15em\lower.25ex\hbox{$\scriptstyle 2$} } = { 2}. 3 8 2 {\text{ min}}} \right) \to\,^{ 1 0 8}{\text{Cd }} + \, \beta^{ - } \left( { 6 2 4 {\text{ keV}}} \right) \, + \, \nu_{\text{e}}^{ - } , \\ & {\text{n }} +\,^{ 10 9}\!{\text{Ag}} \to\,^{ 1 10}\!{\text{Ag }}\left( {T_{\raise.5ex\hbox{$\scriptstyle 1$}\kern-.1em/ \kern-.15em\lower.25ex\hbox{$\scriptstyle 2$} } = { 24}. 5 {\text{ s}}} \right) \to\,^{ 1 10}\!{\text{Cd }} + \, \beta^{ - } \left( { 1 1 8 5 {\text{ keV}}} \right) \, + \, \nu_{\text{e}}^{ - } , \\ \end{aligned} $$

where *T*_½_ is the half-life of particular radio-nuclide.

The average beta particle energies are mentioned in the brackets after the symbol of beta particle.

Indirect products of both above reactions are β^−^ emitters. The beta particles that are the results of silver activation reach the G-M counter and finally produce the electrical impulses which are proportional to the number of neutrons leaving the plasma. Some data are presented in Table 1 by considering both mentioned nuclear reaction as well as their products. The SAC needs to be calibrated by the neutron source with the known neutron yield. The Am–Be or ^252^Cf radioactive neutron sources are both very suitable for the above purpose. The last NSC of the DPF-1000U with the Am–Be source was completed in 2005. There have been some important changes in the device architecture and its surrounding after that. All those changes have improved the DPF-1000U device, however they have influenced on the accuracy of *Y*_n_ assessment.

**Table 1:** Basic nuclear data regarding the nuclear reactions that are engaged in neutron activation monitors

Target	Reaction	Product	*T*_½_^a^	Mean *E*_β_ (keV)	Intensity (%)
^107^Ag	n,γ	^108^Ag	2.382 m	629	95.5
^109^Ag	n,γ	^110^Ag	24.56 s	1,199.36	95.18
^9^Be	n,α	^6^He	806.7 ms	1,567.62	100

				*E*γ (keV)	Intensity (%)
^115^In	n,n’	^115m^ In	4.486 h	336.241	45.8
^115^In	n,γ	^116^In	54.29 m	1,293.56	84.8
				1,097.28	58.5
				416.90	27.2
				2,112.29	15.09
^89^Y	n,n’	^89m^ Y	15.663 s	908.960	99.16

^a^ http://www.nndc.bnl.gov/nudat2/dec_searchi.jsp

Since this time computer simulation based on MCNP calculation was implemented in IPPLM. So during the next NSC of the DPF-1000U device both methods will be used for the assessment of the *Y*_n_.

### Indium monitor of horizontal and vertical distribution of neutrons

Indium occurs naturally on Earth only in two primordial nuclides: ^113^In and ^115^In. Out of these two, abundance of ^115^In is 95.7 %. ^115^In is radioactive, and decays with half-life of 4.41 × 10^14^ years. It is four orders of magnitude larger than the age of the universe and because of that it is considered to be older than the Earth. This situation is uncommon among stable chemical elements. Only indium, tellurium, and rhenium have been shown to have most-abundant isotopes that are radioactive. The less common natural isotope of indium, ^113^In, is stable. Out of all indium radionuclides ^115m^ In and ^116^In have an important meaning for measuring the spatial distribution of neutrons emitted during plasmas experiments performed on DPF-1000U device. These two mentioned isotopes of indium are the result of the following nuclear reactions:

5 $$ \begin{aligned} & {\text{n }} +\,^{ 1 1 5}\!{\text{In}} \to {\text{n}}' \, +\,^{{ 1 1 5\!{\text{m}}}} {\text{In }}\left( {T_{\raise.5ex\hbox{$\scriptstyle 1$}\kern-.1em/ \kern-.15em\lower.25ex\hbox{$\scriptstyle 2$} } = { 4}. 4 8 6 {\text{ h}}} \right) \to\,^{ 1 1 5}\!{\text{In }} + \, \gamma           \quad \left( {E_{\gamma } = { 336}. 2 4 1 {\text{ keV}};I_{\gamma } = { 45}. 8\,\% } \right) \, , \\ & {\text{n }} +\,^{ 1 1 5}\!{\text{In}} \to\,^{ 1 1 6}\!{\text{In }}\left( {T_{\raise.5ex\hbox{$\scriptstyle 1$}\kern-.1em/ \kern-.15em\lower.25ex\hbox{$\scriptstyle 2$} } = { 54}. 2 9 {\text{ m}}} \right) \to\,^{ 1 1 6}\!{\text{Sc }} + \, \beta \, + \, \gamma             \quad\left( {E_{\gamma } = { 1293}. 5 6 {\text{ keV}};I_{\gamma } = { 84}. 8\,\% } \right). \\ \end{aligned} $$

**Fig. 3:**
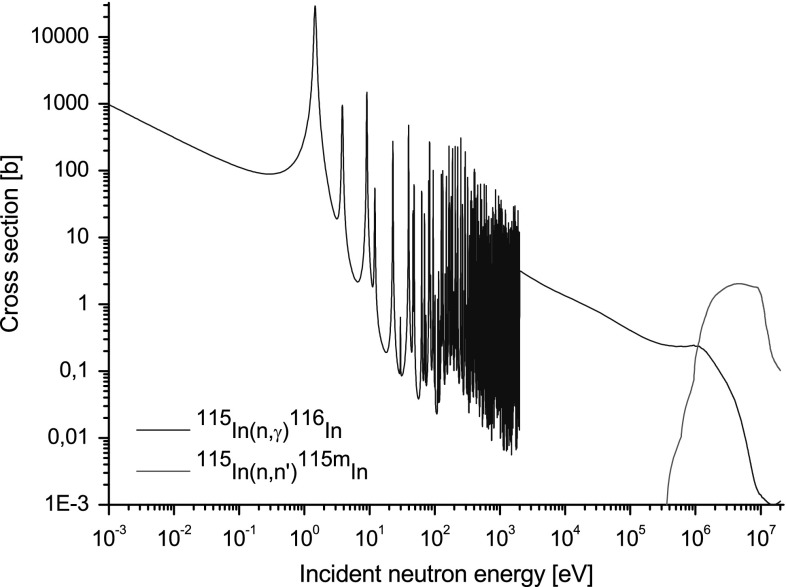
Cross-section for neutron radiative capture (*blue line*) and neutron inelastic scattering (*red line*) with indium nuclei from JEFF 3.1.2. (Color figure online)

The first one is the inelastic scattering reaction induced by neutron with ^115^In nuclei while the second one represents radiative capture of neutrons by the same nuclei of indium. The above mentioned inelastic scattering reaction has the threshold equal to 340 keV (see Fig. 3). It means that this nuclear reaction is not sensitive for neutrons with energy below the threshold. Both mentioned radionuclides of indium are the gamma emitters and thus can be easily detected by gamma spectrometer.

For that reason the gamma spectrometry system based on high purity germanium (HPGe) detector equipped with multichannel analyser (MCA) was used. The detector was provided by the manufacturer with its numerical characterisation and software for mathematical calibration of the system (ISOCS/LABSOCS). This software, uses gamma photon transport calculation, based on the Monte Carlo method, and allows accurate determination of the spectrometer efficiency for many sample shapes, dimensions, and positions in relation to the spectrometer. That allows the elimination of traditional calibration sources, provides significant savings in cost and measurement time. In addition, the flexibility of these tools allows excellent replication of the measured sample geometry resulting in improved accuracy over fabricated calibration source standards. Canberra which is the producer of the above mentioned mathematical calibration software solemnly declares that the typical manufacturing uncertainties for its cause uncertainty equal to ±8–10 % in the detector efficiency response at mid-range (100–400 keV) to high energies. For energies less than 100 keV these deviations can be even worse (around ±15 % for the ^241^Am; 60 keV emission energy).

The information about products of the nuclear reaction explained in Eq. 5 is presented in Table 1.

**Fig. 4:**
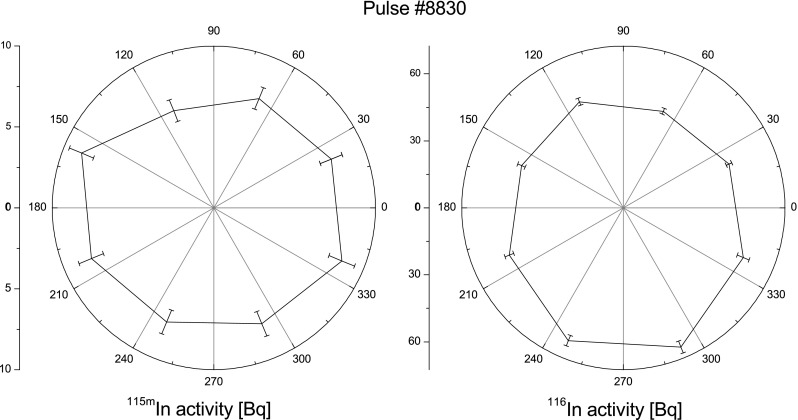
Vertical distribution of two groups of neutrons emitted during the pulse number #8830 performed on DPF-1000U device. Number of total neutrons is expressed as the activity of ^116^In (*right graph*) and the number of neutrons overlapping energy threshold (*E*_n_ = 340 keV) is expressed by activity of ^115m^In (*left graph*)

**Fig. 5:**
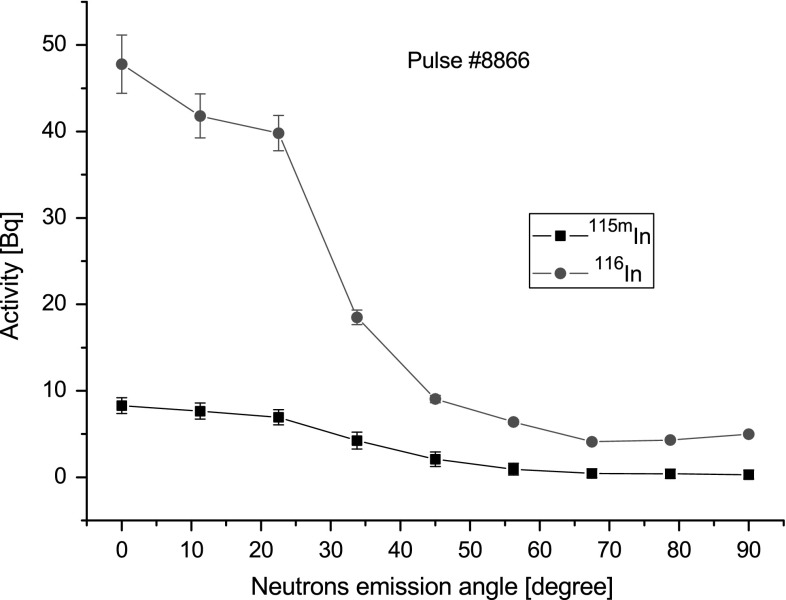
Azimuthal distribution of two groups of neutrons emitted during the pulse number #8866 performed on the DPF-1000U machine. The number of total neutrons is expressed as the activity of ^116^In (*red line*) and the number of neutrons overlapping energy threshold (*E*_n_ = 340 keV) is expressed by activity of ^115m^In (*black line*). (Color figure online)

Based upon nuclear characteristic of indium the neutrons angular distribution experiment has been conducted on DPF-1000U facility [5]. The indium sample geometry has been the subject of optimization studies [6]. Optimisation is a process of finding the best sample shape among plenty of others and the biggest sample mass. During that process, two phenomena have been considered commonly, the activation of indium by the neutrons from plasma experiment and measurability of their activation products by means of gamma spectrometry method. Fulfilling the above mentioned criteria, the heavy cylindrical sample with diameter of 63.7 and 4.3 mm in thickness was involved as the monitor of angular (horizontal and vertical) distribution of neutrons from plasmas experiments. The set of massive indium samples has been mounted on the wall of the DPF-1000U device in two different ways. The first set of eight samples mounted around the cylindrical wall of the device, on the layer crossing the place of plasma formation allows to measure the vertical distribution of two groups of neutrons: overlapping the energy threshold and all neutrons (the results of the above experiment are shown in Fig. 4). The second set of nine samples mounted along the cylindrical wall of the device allows for the measurement of the horizontal distribution of the mentioned group of neutrons. The results are shown in Fig. 5.

This kind of experiment was useful for the estimation of an anisotropy of neutrons which is one of the crucial parameter for understanding plasmas formation on the DPF-1000U machine. All indium activation experiments performed with the DPF-1000U device has been supported by MCNP calculations [7]. For the estimation of an anisotropy the useful were only the values of the activity of threshold reaction product i.e. ^115^In.

### Yttrium monitor for neutron emission yield

Yttrium has only one naturally occurring stable isotope ^89^Y. The following nuclear reaction is considered:

6 $$ {\text{n }} +\,^{ 8 9}\!{\text{Y}} \to\,^{{ 8 9{\text{m}}}}\!{\text{Y }}\left( {T_{\raise.5ex\hbox{$\scriptstyle 1$}\kern-.1em/ \kern-.15em\lower.25ex\hbox{$\scriptstyle 2$} } = { 15}. 6 6 3 {\text{ s}}} \right) \, + {\text{ n}}' \, + \, \gamma             \quad \left( {E_{\gamma } = { 9}0 8. 9 6 {\text{ keV}};I_{\gamma } = { 99}. 1 6\,\% } \right). $$

**Fig. 6:**
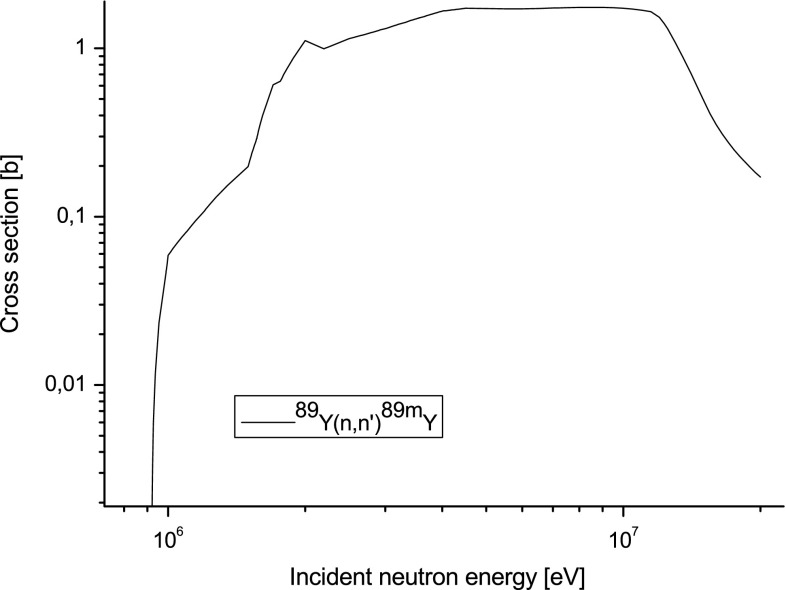
Cross-section for inelastic scattering of neutrons with ^89^Y nuclei from JEFF 3.1.2

^89m^Y is the relatively short lived radionuclide and because of that it is very useful as the neutron monitor for repetitive plasma devices. FNYM does not need any neutron moderator to allow neutrons detection. Neutron monitoring is realized by simple counting of gammas emitted from activated meta state of yttrium isotope.

Nuclear reaction of inelastic scattering of neutrons with yttrium nuclei has broad perspective to be implemented as the FNYM in plasma experiments. It is the threshold reaction (see Fig. 6) that means it is mostly sensitive for primary fusion neutrons.

During the preliminary experiment, the yttrium sample was put on the DPF-1000U surface before each discharge. Its position was situated on the 90° to the main axis of the DPF-1000U device and on the plain crossing the plasma focus. Yttrium was activated during discharge, than removed manually and put to the shielding house, standing close to the device, and measured. The removal of the sample took usually up to 20 s. The activity of ^89m^Y (expressed in Bq) was estimated by the MCA software for the particular discharge time. The measurement of yttrium sample activity was performed with gamma spectrometry system equipped with HPGe detector. The efficiency calibration of the spectrometry system was done by the software (ISOCS/LABSOCS) attached to the system. After each measurement the yttrium sample was put again in the same position on the device wall. Using the same sample was allowed because the repetition time of the DPF-1000U device is approximately 20 min. This means that all activated nuclei disintegrated and turned themselves to the ground state. This experiment shown that the activity induced in yttrium sample is directly proportional to the *Y*_n_ monitored by SAC.

**Fig. 7:**
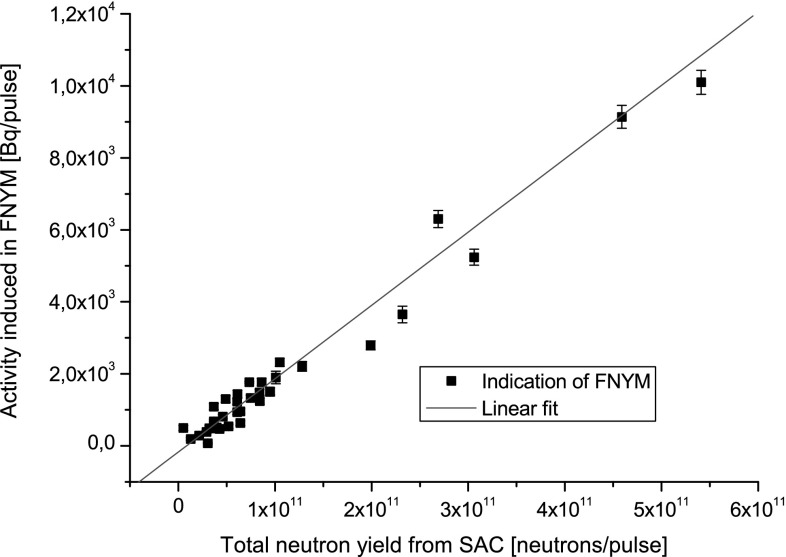
Activity of ^89m^Y induced in FNYM versus neutron strength expressed as indication of SAC observed during dozen of pulses performed at DPF-1000U device

The information of half life of the ^89m^Y and its gamma quanta energy is presented in Table 1. The results of the measurements of activity induced in FNYM as the function of *Y*_n_ measured by SAC are shown in Fig. 7.

### Beryllium counter

Beryllium has 12 known isotopes, but only one of these isotopes ^9^Be is stable and a primordial nuclide. As such, beryllium is considered a monoisotopic element. It is also a mononuclidic element, because its other isotopes have such short half-lives that none are primordial and their abundances are very low. ^9^Be is very promising as an activation material. The following nuclear reaction is taken into account:

7 $$ {\text{n }} +\,^{ 9}\!{\text{Be}} \to\,^{ 6}\!{\text{He }}\left( {T_{\raise.5ex\hbox{$\scriptstyle 1$}\kern-.1em/ \kern-.15em\lower.25ex\hbox{$\scriptstyle 2$} } = \, 0. 80 7 {\text{ s}}} \right) \to\,^{ 6}\!{\text{Li }} + \, \beta^{ - } \left( { 1 5 6 8 {\text{ keV}}} \right) \, + \, \nu_{\text{e}}^{ - } . $$

**Fig. 8:**
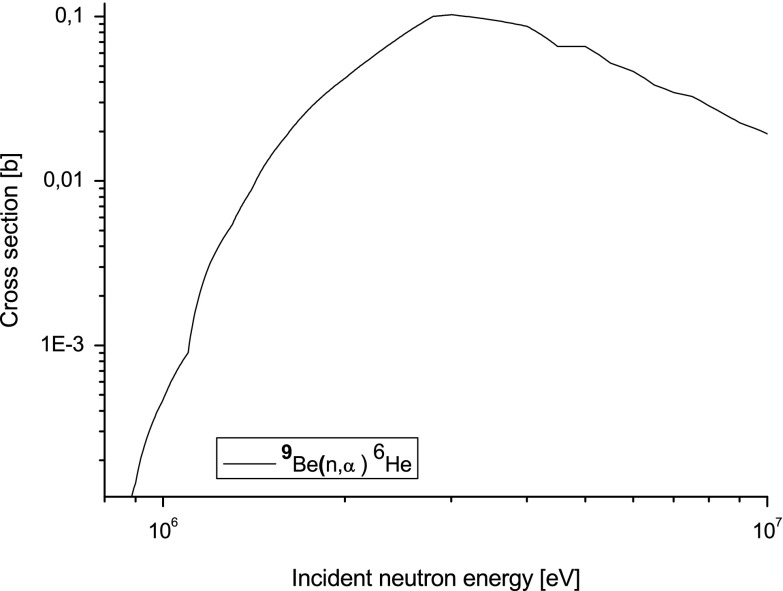
Cross-section for particular transition with emission of alpha particle from beryllium nuclei from JEFF 3.1.2

The cross-section for that reaction is shown in Fig. 8. It has a useful threshold near 1 MeV, which means that undesirable scattered neutrons do not undergo that nuclear reaction and therefore are not measured.

Large area gas sealed proportional detector has been chosen as β^−^ particles counter. Beryllium plate (GoodFellow made, 99.0 % purity), adjoins the detector and is centred in relation to the window.

The calibration of the BNAC consists of several steps: measurements with radioactive β^−^ and neutron calibration sources and a number of various Monte Carlo calculations of β–particle and neutron transport as well. The MCNP5 [7] Monte Carlo code with MCNP5DATA [8] cross section library have been used for above- mentioned calculations. During the first step of the calibration the flat ^90^Sr/^90^Y calibration source has been chosen as the sufficient source of β^−^ particles. The β^−^ particles which deposit in SP-126C detector their energy have been calculated by means of MCNP. Comparison of ^90^Sr/^90^Y source measurements with above-mentioned MCNP calculations allowed determining a calculation to experiment ratio which has been applied in following calculations.

The next step of the calibration considered MCNP simulation of the beryllium response to the particular neutron source in strictly defined measuring geometry. Taking into account the experimental conditions the suitable MCNP neutron calculation has been performed for particular location of the detector regarding DPF-1000U device. Additionally, the ENDF/B-VII.0 nuclear cross-section library for ^9^Be(n,α)^6^He reaction has been implemented and the rate of that nuclear reaction per one source neutron has been obtained.

Taking into account the results of the calibration of the proportional detector as a β^−^ particles counter and calculated nuclear reaction rate, the calibration coefficient was estimated. It allowed the determination of the *Y*_n_ from the number of β^−^ particles counts.

**Fig. 9:**
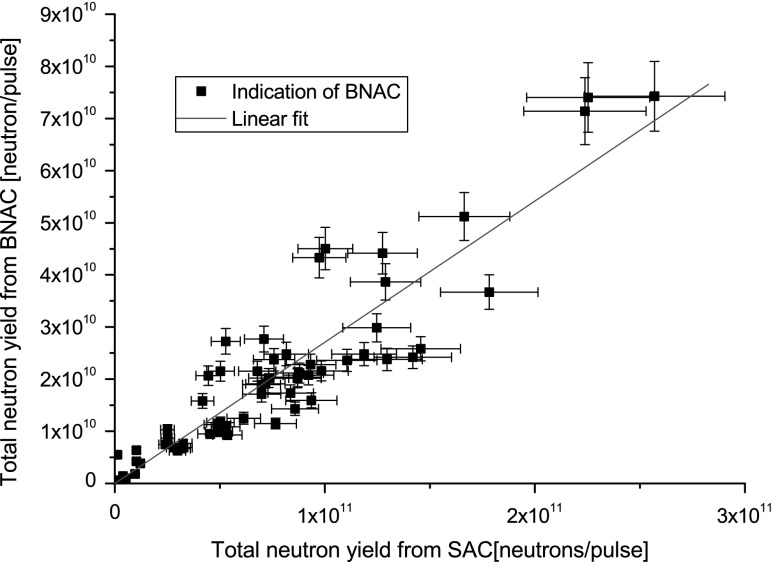
Indication of BNAC (*black squares*) is a linear function (*red line*) of *Y*_n_ from SAC. (Color figure online)

BNAC designed to be operated on DPF-1000U has proved its utility during a number of experiments with results well correlated with SACs. These data are shown in Fig. 9. It is also shown that *Y*_n_ estimated by means of BNAC is not exactly equal to the obtained by SAC. It is probably due the changes made in the DPF-1000U facility architecture.

## Conclusions

The dense magnetized plasma that is generated inside the DPF-1000U device has to be diagnosed and a few different types of metrological methods are implemented for the realization of the above task. Among others the activation method occupies an important position. The activation of silver foil uses the radiative capture reactions is the oldest method for monitoring the neutron strength, and SAC has been used among plenty of plasma experiments. This method needs to be calibrated by means of separate neutron source. The important disadvantage of silver activation monitor is its high sensitivity to the scattered neutrons. Both BNAC and FNYM use threshold reaction therefore they are not sensitive to scattered neutrons like radiative capture reaction. The relatively short half-lives of the radionuclides occurring in mentioned nuclear reactions are the great advantage of both methods. Plasma diagnostics based on the BNAC and FNYM methods can be cross-calibrated with the use of MCNP calculation including previously calibrated methods. ^115m^In meta state that has a relatively long half-life is useful for researching the angular neutron distribution: horizontal and vertical. Thus the above mentioned radionuclides obtained in result of plasma neutron interaction with their primordial nuclides are very useful for plasma diagnostics.
